# Bronchoscopy and Lung Fine-Needle Aspiration for Antemortem Evaluation of Pulmonary Involvement in Dogs with Naturally Occurring Canine Leishmaniosis

**DOI:** 10.3390/pathogens11030365

**Published:** 2022-03-17

**Authors:** Ioannis Kavarnos, Dimitra Pardali, Georgia D. Brellou, Elias Papadopoulos, Maria Kritsepi-Konstantinou, Katerina K. Adamama-Moraitou

**Affiliations:** 1 Companion Animal Clinic (Medicine Unit), School of Veterinary Medicine, Faculty of Health Sciences, Aristotle University of Thessaloniki, 54627 Τhessaloniki, Greece; gianniskavarnos@hotmail.com (I.K.); kadamama@vet.auth.gr (K.K.A.-M.); 2 Diagnostic Laboratory, School of Veterinary Medicine, Faculty of Health Sciences, Aristotle University of Thessaloniki, 54627 Thessaloniki, Greece; dpardali@vet.auth.gr (D.P.); mkritsep@vet.auth.gr (M.K.-K.); 3 Laboratory of Pathology, School of Veterinary Medicine, Faculty of Health Sciences, Aristotle University of Thessaloniki, 54627 Thessaloniki, Greece; mprellou@vet.auth.gr; 4 Laboratory of Parasitology and Parasitic Diseases, School of Veterinary Medicine, Faculty of Health Sciences, Aristotle University of Thessaloniki, 54124 Thessaloniki, Greece

**Keywords:** canine leishmaniosis, bronchoscopy, bronchoalveolar lavage, bronchial mucosa biopsies, immunohistochemistry, immunocytochemistry, needle aspiration, IFAT, *Leishmania infantum*

## Abstract

Clinical manifestations from the lower respiratory tract are rare in canine leishmaniosis (CanL), making bronchoscopy and lung fine-needle aspiration (FNA) seldomly justified. The aim of this prospective study was to investigate the involvement of *Leishmania infantum* in the lungs of dogs with naturally occurring CanL by bronchoscopy and examination of bronchoalveolar lavage fluid (BALF), bronchial mucosa biopsies, and FNA, using immunodiagnostics. Dogs with relevant concurrent diseases and azotemia were excluded. Cough was detected in 5/31 (16.1%) dogs. Lesions (hyperemia, edema, mucosal granularity, secretions) were identified upon bronchoscopy in 19/31 (61.3%) dogs. The cytology of BALF revealed histiocytic inflammation in 14/31 (45.2%) dogs; the parasite was identified in one dog (3.2%). The immunofluorescence antibody test in BALF was positive in 15/31 (48.4%) dogs. Histopathology of bronchial mucosa and/or adjacent alveoli revealed lesions (mononuclear cell infiltration, fibrosis, edema, thickening of the inter-alveolar septa) in 24/31 (77.4%) dogs, with no *Leishmania* amastigotes. Positive antigen staining was observed within the cytoplasm of mononuclear cells in immunocytochemistry and immunohistochemistry. Μononuclear cells showed antigenic positivity in bronchial mucosa (27/31; 87.1%), BALF (30/31; 96.8%), and lung FNA (27/31; 87.1%). In conclusion, lungs seem to be affected from CanL more commonly than previously believed, and bronchoscopy allows obtaining valuable samples for antemortem diagnosis.

## 1. Introduction

Canine leishmaniosis (CanL) is a multisystemic, vector-borne zoonosis, which is highly endemic in the Mediterranean region and is caused by *Leishmania infantum* (syn. *L. chagasi*). The outcome of the infection ranges from local elimination of the parasite to parasite dissemination and development of mild to severe clinical signs and/or clinicopathological abnormalities. Cells of the mononuclear phagocytic system, neutrophils, eosinophils, hepatocytes, endothelial and dendritic cells, and fibroblasts can be infected by *Leishmania* parasites; therefore, a variety of different organs can be affected, justifying the clinical polymorphism of the disease [1,2,3].

Weight loss, lethargy, generalized lymphadenomegaly, skin and ocular lesions, onychogryposis, polyuria, polydipsia, splenomegaly, and lameness are some of the most typical clinical findings in symptomatic dogs [4,5,6]. Mild to moderate nonregenerative anemia, hyperglobulinemia, proteinuria, and renal azotemia are the most common laboratory abnormalities in dogs with CanL [7]. Cases of intestinal [8], bone [9], pancreatic [10] central nervous system [11], and lung [12,13,14,15,16,17,18,19] involvement have been reported less frequently.

Pulmonary involvement in human visceral leishmaniosis (VL) is considered rare; however, it has been described since 1959 [20] by several authors [21,22,23]. Clinical signs originating from the lower respiratory tract are considered rare in VL cases. Nevertheless, dry cough was noted in 16% and 25.9% of the cases in two retrospective studies [24,25], and in separate case reports [26,27,28,29]. Bronchial mucosa involvement has been described in immunocompetent [26,30], immunocompromised [28,29,31], and bronchial asthma patients [32]. In all cases, bronchoalveolar lavage fluid (BALF) and bronchial mucosal biopsies were obtained via bronchoscopy and led to the diagnosis of VL.

The parasitism of the lungs of dogs with CanL was first documented in 1937 [33]. Since then, a number of case reports, as well as retrospective and experimental studies, suggest that pulmonary involvement is an existing entity in CanL [12,13,14,19,34,35]. Nevertheless, respiratory signs are considered rare and still not described in detail [4,34,36]. Histopathological pulmonary alterations indicating chronic diffuse interstitial pneumonia in CanL are described in several studies and case reports, with frequency varying from 77% to 100% [12,13,14,15,17,37]. Interstitial pneumonitis has been reported as a common finding, noted in 80.5% of the cases (33/41) [13]. Thickening of the inter-alveolar septa is the most prominent finding [13,14,15,16,17,37], which has been confirmed by morphometry [12], quantitatively estimating the difference between infected and healthy dogs. Diffuse or focal inflammatory lesions of the alveolar septa are composed of mononuclear cells, predominantly macrophages, lymphocytes, and plasmacytes [12]. Fibrosis is another common histopathological finding, described in the vast majority of the cases studied. Compared to healthy dogs, lung collagen deposition is also increased in dogs with CanL [12,37]. *Leishmania* parasites are rarely identified in lung tissue by routine histopathology [12,14,18,34] or cytology [15], despite the presence of compatible lesions. Immunohistochemistry provides better results in detecting the parasite and has the additional advantage of identifying it as the cause of the lesions observed [13,14,15,37].

The infrequent pulmonary signs of dogs with CanL in the clinical setting have understated bronchoscopy and fine-needle aspiration (FNA) of the lung as diagnostic tools in cases of CanL. To the authors’ knowledge, there are no studies where bronchoscopy and examination of BALF and FNA samples of the lung have been used for diagnostic purposes in dogs with naturally occurring CanL. The purpose of this study was to investigate the presence of *L. infantum* and the associated lesions in the lungs of dogs with naturally occurring CanL by bronchoscopy and examination of BALF, bronchial mucosal biopsies, and FNA samples, using immunofluorescence antibody test (IFAT), cytology, histopathology, immunocytochemistry and immunohistochemistry, as well as to investigate the value of these techniques for the diagnosis and respiratory status evaluation.

## 2. Results

### 2.1. Study Population

Thirty-one dogs fulfilled the inclusion criteria and were enrolled in the study.

Mild, unproductive cough was noted in 5/31 (16.1%) dogs during tracheal palpation, while none of the 31 dogs had abnormal findings upon thoracic auscultation. No statistical difference (*p* > 0.05) was recorded between the sex of the dogs and the presence of cough. Most of the dogs of the study population belonged to disease stage 2 (14/31; 45.2%) and 3 (13/31; 41.9%), while 3/31 (9.7%) and 1/31 (3.2%) dogs belonged to stages 1 and 4, respectively (Table 1 and Table 2).

### 2.2. Clinicopathological Examination

Cytological examination of lymph nodes was positive for *Leishmania* amastigotes in all study dogs, while bone marrow smears were positive in 29/31 (93.5%) dogs.

Serum IFAT was positive in all dogs included in the study and varied from 1/100 to >1/3200, while BALF IFAT was positive in 15/31 (48.4%) dogs.

Radiography of the thorax revealed lesions in 12/31 (38.7%) dogs. Bronchial and interstitial patterns were identified in 4/12 (33.3%) and 5/12 (41.6%) dogs, respectively, while 3/12 (25%) of them had mixed bronchial and interstitial radiographic signs. Lesions were diffuse, except for 2/5 (40%) dogs with interstitial patterns, which were focal.

### 2.3. Bronchoscopy, Lung FNA, Immunodiagnostics

**Figure 1:**
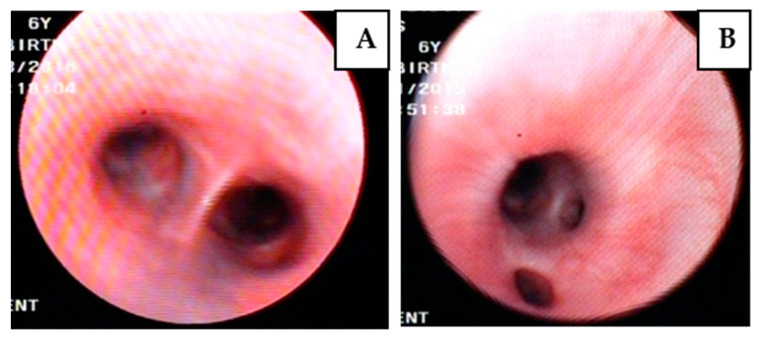
Hyperemia (**A**) and edema (**B**) seen upon bronchoscopy of two dogs with leishmaniosis.

Bronchoscopy revealed lesions in 19/31 (61.3%) dogs (Figure 1, Table 3). Table 4 shows the categorization of the dogs according to the severity of their bronchoscopic lesions. The majority of the dogs were allocated to the mild bronchoscopic group.

**Figure 2:**
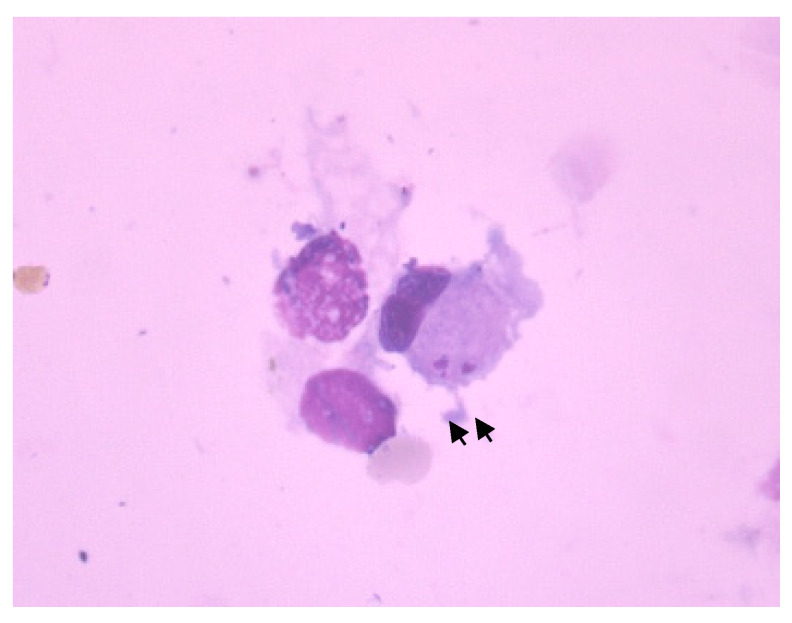
*Leishmania* amastigotes (arrows) in a macrophage in bronchoalveolar lavage fluid cytology (Wright–Giemsa stain, 100×).

Cytological examination of BALF revealed inflammation in 14/31 (45.2%) dogs. Histiocytic inflammation was the most common finding (9/14; 64.3%) followed by pyogranulomatous inflammation (5/14; 35.7%). None of the cytological smears was hemorrhagic, and *Leishmania* amastigotes were identified in one dog (3.2%) (case number 25) (Figure 2).

On cytological examination of lung FNA smears, no *Leishmania* amastigotes were found in all 31 dogs of the study.

**Figure 3:**
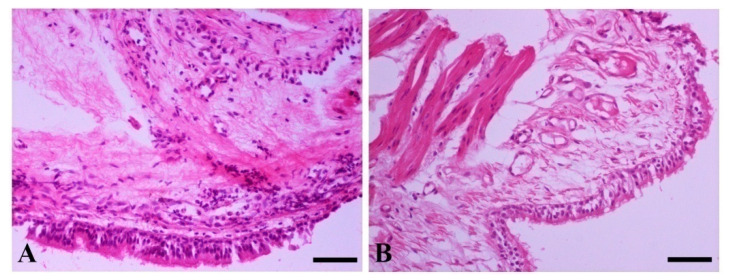
Histopathological sections from bronchial biopsies of two dogs with leishmaniosis: (**A**) inflammatory cell infiltration consisting mostly of mononuclear cells and neutrophils, intermingled with fibroblasts, is obvious in the lamina propria; (**B**) bronchial mucosa is characterized by intraepithelial and lamina propria edema with vascular distension. H&E, bar = 50 μm.

Histopathological examination of bronchial mucosa biopsies revealed pathological changes in 24/31 (77.4%) dogs, most of them being of mild severity (Table 5 and Table 6). In addition to bronchial mucosa, adjacent alveoli were also included in the specimens obtained in 4/31 (12.9%) cases, and thickening of the inter-alveolar septa was observed in all of them, due to mononuclear cell infiltration (consisting mostly of macrophages and lymphocytes) and fibrosis, with (1/4; 25%) or without (3/4; 75%) edema (Figure 3). The sex of the dogs had no effect on the histopathological changes (*p* > 0.05).

**Figure 4:**
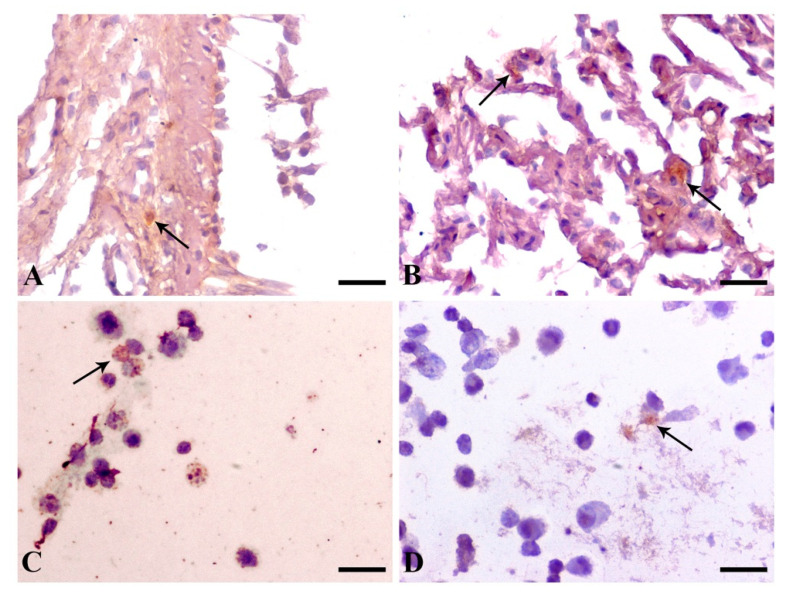
Representative *Leishmania major* surface protease gp63 (clone 96–126) immunohistochemistry in bronchial mucosal biopsies (**A**) and (**B**), as well as immunocytochemistry in BALF (**C**) and lung fine-needle aspiration smears (**D**) showing positive staining. (**A**) Bronchial mucosal biopsy: a macrophage in the thickened lamina propria showing positive gp63 antigen immunolabeling (arrow). (**B**) Bronchial mucosal biopsy containing adjacent alveoli: diffuse cytoplasmic staining is prominent in interalveolar macrophages (arrows). (**C**) BALF: characteristic *Leishmania* amastigotes appear positively stained in the cytoplasm of macrophages (arrow). (**D**) FNA: a few positively stained amastigotes are present in a macrophage (arrow). DAB chromogen, hematoxylin counterstain. (**A**,**B**) Bar = 25 μm. (**C**,**D**) Bar= 18.75 μm.

Immunohistochemical staining for gp63 antigen revealed positive cytoplasmic immunoreactivity in mononuclear cells of bronchial mucosal biopsies in 27/31 (87.1%) dogs. Additionally, in BALF and lung FNA smears, mononuclear cells were positively stained in 30/31 (96.8%) and 27/31 (87.1%) dogs, respectively (Figure 4).

## 3. Discussion

The results of this study coincide with the findings of previous studies performed exclusively in postmortem cases that lungs can be affected by *Leishmania infantum*. The parasite can be hard to detect using traditional cytological and histopathological examination; however, immunodiagnostics on lung mucosal biopsies, BALF, and lung FNA smears show superior sensitivity. The bronchial mucosal lesions identified endoscopically and histopathologically are common, yet mild and are seldomly associated with significant clinical manifestation.

Respiratory signs were an uncommon clinical manifestation, as cough was only present in 5/31 (16.1%) cases. This comes in agreement with the literature, as Koutinas et al. [4] reported respiratory signs as clinical complaints in 3/158 (1.9%) cases and Slappendel [6] noted cough in 5/95 (6%) and pneumonia in only 2/95 (1.5%) dogs. However, neither of them clarified whether the respiratory symptoms were attributed to CanL or comorbidities.

Our study population mainly consisted of dogs with stage 2 and 3 CanL, probably due to the exclusion criterion of azotemia, considering the fact that CanL very often causes chronic kidney disease, especially at later stages. Azotemia can be associated with pulmonary changes compatible with pneumonitis that may be falsely attributed to leishmaniosis [12,38,39] and also carries greater anesthesia risk; therefore, azotemic dogs were excluded from the study. Dogs with stage 1 CanL have mild disease that can go undetected by the owner, which is the most likely reason why they were underrepresented with only three included in this study. High parasitic load is associated with more intense immune complex formation, greater serum IFAT titers, and more severe clinicopathological manifestations of the disease [2,3,40] Due to the exclusion criteria of the present study, we only included one dog with stage 4 CanL. Consequently, the lower parasite population in lung tissue and, therefore, milder lesions could be justified by the milder cases enrolled in the study.

Cytological examination of lymph node smears showed marginally greater sensitivity compared to bone marrow. This could be explained by hemodilution of bone marrow samples [40].

As expected, serum IFAT titers were positive in all 31 dogs, a fact that is true in the vast majority of dogs with symptomatic CanL [41]. Noteworthily, almost half of the dogs showed positive BALF IFAT and positive serum IFAT, which could be the result of immune response of the lungs, reflecting their susceptibility to the disease in certain dogs [2]. Local production of immunoglobulins in the lungs has been previously demonstrated in BALF, in human patients with fungal, bacterial, and allergic lung conditions [42,43,44] but never before in dogs with CanL. Dilution of the BALF to a variable and unpredictable degree during sampling may have had a negative impact on the positivity of IFAT.

Radiographic lesions were identified more often than reported in the human literature [45]. This may be attributed to chronic bronchopulmonary disease, caused by unknown, previous medical conditions, or to the more chronic course of the disease in dogs, compared to humans who presented with acute and severe respiratory symptoms.

Bronchoscopy and cytological examination of BALF in most cases of our study indicated chronic, mild, and diffuse inflammation of the bronchial mucosa, while *Leishmania* amastigotes were cytologically identified in only one case. Comparable findings can only be extracted from human medicine, where bronchial involvement has been sparsely reported [26,30,32,46]. Kotsifas et al. [26] described mild and diffuse redness throughout the bronchial tree, with inflammation and a polypoid lesion in an immunocompetent patient with cough and episodes of hemoptysis. Similar bronchoscopic findings with inflammation and swollen bronchial mucosa with a tumor like lesion were reported by Robibaro et al. [32] in a patient with exacerbating asthma. In this case, *Leishmania* amastigotes were identified in few macrophages, in BALF cytological examination. Diffuse bronchial mucosa inflammatory changes were also noted in a case of *Leishmania donovani* infection in a patient with cough, dyspnea, and hemoptysis [30]. Jokipii et al. [29] identified *Leishmania* amastigotes in BALF cytological smear from an immunocompromised patient presenting with cough and fever. The authors begged the question of the value of BALF in diagnosing lung involvement in visceral leishmaniosis.

Diagnostic FNA of the lung has been previously reported in a patient with VL who presented with lung nodules and severe respiratory distress [46]. The procedure was CT-guided and numerous *Leishmania* amastigotes were identified during cytology. The presence of the lung nodules and the precision provided by the guidance of the CT presumably increased the sensitivity of the method, compared to our study.

Histopathological lesions compatible with chronic inflammation of the bronchial mucosa were observed in the majority of our population, while interstitial pneumonitis was also identified in cases where alveoli were included. This comes in agreement with the current literature, where such lesions have been described in up to the total of the study population in necropsied dogs [12,13,15,17,18]. The distribution is usually diffuse and of mild severity, probably reflecting the chronic course of the disease, since the dogs, similarly to our study, were naturally infected. However, this was the first report that all diagnostic procedures were performed antemortem, via bronchoscopy, obtaining significantly smaller tissue specimens compared to those taken at necropsy. As a consequence, the samples consisted predominantly of bronchial mucosal tissue; however, in a small number of cases, adjacent alveoli were also obtained, showing thickening of the inter-alveolar septa. The above cases presented concurrent lesions in the bronchial mucosa. Parasite amastigotes were not identified during histopathology, confirming that *Leishmania* parasites are seldomly demonstrated even when lesions are present [12,13,15,17,18].

Immunohistochemical examination of bronchial mucosa biopsies and immunocytochemical examination of lung FNA smears revealed parasitism in the vast majority of the cases, despite the lack of visual identification of the amastigotes using optical microscopy. Sensitivity of immunohistochemistry ranged widely in previous studies, from 0% to 20.8% [15,17,18]. Additionally, immunocytochemistry of BALF was the most sensitive diagnostic method in our study, with only one negative result, despite the inability to identify amastigotes in cytologic examination. This superiority of immunodiagnostics could be attributed to the ability to detect even fragmented antigenic particulates, while histopathology and cytology are restricted to intact parasites. Pulmonary macrophages’ increased microbicidal ability and local destruction of the parasites may be the cause of the low numbers of intact parasites [12], making immunodiagnostics the diagnostic method of choice when lung involvement is suspected.

Study limitations include the limited number of dogs included due to the exclusion criteria. Most dogs with very severe stage 4 CanL that would be expected to carry the largest parasitic load were excluded from the study, due to azotemia; therefore, our results may be downscaled. Moreover, bronchial mucosa specimens obtained via bronchoscopy were considerably smaller compared to fragments obtained during necropsy and may have limited the diagnostic power of histopathology and immunohistochemistry.

## 4. Materials and Methods

### 4.1. Study Population

Dogs presented to the Companion Animal Clinic (Unit of Medicine), School of Veterinary Medicine, Aristotle University of Thessaloniki through 2015–2017 and diagnosed with CanL were eligible candidates for this prospective study. Diagnosis was based on compatible physical examination findings and/or clinicopathological abnormalities, combined with positive serological testing (IFAT) and/or parasitological identification of *Leishmania* amastigotes in cytological smears of the lymph nodes or bone marrow. Additionally, canine dirofilariasis and conditions that could cause pulmonary abnormalities, such as renal azotemia and infectious or neoplastic respiratory disease, were considered exclusion criteria. Dogs that had received antileishmanial drugs during the past 6 months were also excluded from the study. Signalment, history, and physical examination findings were recorded from all the dogs of the present study.

### 4.2. Clinicopathological Examination

Venus blood samples were collected after jugular venipuncture for hematology and routine biochemistry analysis. Adequate hydration status was necessary to estimate serum creatinine and urea nitrogen concentration. A commercially available serological ELISA-based test (IDEXX 4Dx Plus Test, IDEXX Laboratories, Westbrook, ME, USA) was used to detect *Dirofilaria immitis* (heartworm) antigen, while IFAT was performed for the detection of anti-leishmanial antibodies and determination of *Leishmania* titer in serum of all cases. The cutoff value for positivity in IFAT was established at 1:100. Urinalysis was conducted in samples collected via cystocentesis. Fine-needle aspiration of the palpable lymph nodes and bone marrow aspiration was performed, and cytological smears were prepared and were stained with Wright–Giemsa stain. Cytological examination of lymph nodes and bone marrow cytology smears was performed. On the basis of physical examination findings and clinicopathological abnormalities and in order to assess the severity of the disease, dogs were classified into four groups (stages 1 to 4) according to grading system. Plain left and right lateral, as well as dorsoventral, radiographs of the thorax were obtained with the dogs under general anesthesia.

### 4.3. Bronchoscopy, Lung FNA, Immunodiagnostics

Fiberoptic bronchoscopy was then performed with the use of a sterilized 5.0 mm diameter BF-PE2 Olympus flexible endoscope. Bronchoscopic appearance of the bronchial mucosa was evaluated, and the presence and severity of secretions and of any lesions (hyperemia, edema, mucosal granularity, etc.), along with their severity and distribution, were recorded. A modified scoring system by Thompson was used.

Hyperemia and edema of the bronchial mucosa were scored on a scale from 0 to 3, depending on the presence and severity of the lesions for each dog, while secretions and mucosal granularity were scored with one point if present and no points if absent. Depending on the extent of each lesion, one or two points were given for localized or diffuse distribution, respectively, and the total endoscopic score was calculated by adding all the points given to describe the overall bronchoscopic appearance of the bronchial mucosa of each dog. The bronchoscopic score ranged from 0 (normal) to a maximum of 16 and was used to form four groups on the basis of the severity of the endoscopic lesions: score 0 (normal), score 1–5 (mild), score 6–10 (moderate), and score 11–16 (severe).

After evaluation of the bronchial mucosa was completed, two syringes were pre-drawn with sterile saline. Bolus sterile saline of 0.5–1 mL/kg, but no more than 15 mL, was instilled after wedging the tip of the bronchoscope in a small bronchusof the right caudal lung lobe, depending on the length and diameter of our instrument and the size of the dog, and then aspirated. The procedure was repeated for the left caudal lobe. The recovered BALF was collected in sterile tracheal suction containers (PharmaPlast, Kafr-elZayat, Egypt) and placed in a sterile tube for culture and an Eppendorf for IFAT. IFAT was carried out by applying the same method as for serum samples, but without estimating the exact titer. Furthermore, a separate aliquot was used to produce cytocentrifuge slides for cytological evaluation, and another was centrifuged at 350× *g* for 10 min; the sediment was transferred into a BD centrifuge tube filled with 10 mL of CytoRich Red™ Preservative Reagent fluid (BD–Tripath Imaging, Burlington, North Carolina, USA) for immunocytochemistry. The latter tube was centrifuged at 350× *g* for 10 min; the supernatant fluid was discarded, and the sediment was diluted again with 10 mL of distilled water. The tube was then centrifuged again at 350× *g* for 5 min; the supernatant fluid was discarded. A portion of 200 μL of the sediment was then transferred in the BD Settling Chamber that was fitted on the top of the SurePath™ precoat slide (BD–Tripath Imaging, Burlington, NC, USA) and was left for 10 min, for the sedimentation to complete. After adding 600 μL of an alcoholic mixture made up of ethanol (Dehyoladsolute, Bio-optica, Milano, Italy) and resting for 1 min, the supernatant was decanted, and the slide with the settling chamber was turned upside down on absorbable paper and left for 1 min to dry. The settling chamber was then removed, and the slide was submitted for immunocytochemistry.

After completion of the BALF procedure, 3–4 bronchial mucosal biopsy samples were obtained. Sampling location was based on distribution of the lesions and local vascularity. Biopsy specimens were fixed in 10% neutral buffered formalin and then submitted for histopathological and immunohistochemical evaluation. Paraffin-embedded tissue blocks were cut into 5 µm sections and stained with hematoxylin and eosin (H&E). Blinded histopathological and immunohistochemical assessment was conducted by a single pathologist (G.D.B.). Tissue sections were semi-quantitatively evaluated for the following lesions: inflammatory cell infiltration, fibrosis, and edema of bronchial mucosa. Lesions were assigned to a category showing an ordered progression in severity: 0—normal, 1—mild, 2—moderate. Bronchial mucosa was also examined for absence—0 or presence—1 of bronchial gland hyperplasia and thickening of vessel walls and bronchial smooth muscles. Lastly, when alveoli were available, inter-alveolar septa were examined for absence—0 or presence—1 of inflammatory cell infiltration, fibrosis, and edema. Additionally, immunohistochemistry was performed on serial tissue sections, using the Super-Sensitive Polymer-HRP IHC Detection System (BioGenex). After deparaffinization and rehydration, heat-induced antigen retrieval was performed with EDTA (ethylenediaminetetraacetic acid) buffer, pH 9 for 25 min at 95 °C. Sections were incubated with a *Leishmania major* surface protease gp63 monoclonal antibody (Clone 96/26, Cedarlane Laboratories, Burlington ON, Canada) overnight at 4 °C, followed by poly-HRP reagent (from Super Sensitive Polymer-HRP IHC Detection system). Color was developed with a DAB substrate–chromogen system (Biogenex, Fremont, CA, USA), and tissues were counterstained with hematoxylin. Lastly, dogs, while still under general anesthesia, were placed in right recumbency, and blind lung FNA using a 1.5 inch 22-gauge spinal needle was performed after aseptic preparation of the seventhto ninthintercostal space (Taylor in small animal clinical techniques 2016). Cytological smears were prepared, and the procedure was repeated. This time, the content of the needle was expelled in a tube filled with 10 mL of CytoRich Red™ Preservative Reagent fluid (BD–Tripath Imaging, Burlington, NC, USA), for immunocytochemistry. After the end of the procedure, the dogs were placed in left lateral recumbency.

Immunocytochemistry was applied toBALF and lung FNA smears. The procedure was performed as described for the immunohistochemical staining above, but steps for deparaffinization and rehydration were omitted, and slides were washed three times in phosphate buffer saline. All dogs recovered uneventfully from the diagnostic procedures and anesthesia.

### 4.4. Statistical Analysis

All statistical analyses were performed using SPSS v23 (IBM, New York, NY, USA). Statistical significance was set at the 0.05 level. We performed *t*-tests to compare the different groups.

## 5. Conclusions

To the authors’ knowledge, this is the first study to use in vivo diagnostics to assess lung involvement in dogs with naturally occurring CanL. Traditional methods such asserology and lymph node cytology remain the diagnostic methods of choice for diagnosing CanL; nevertheless, bronchoscopy shows great potential, allowing inspection of the bronchial mucosa and the obtention of biopsies and BALF. Immunodiagnostics of bronchial mucosa biopsies, BALF, and lung FNA smears are effective methods to investigate lung involvement in CanL cases. Interstitial pneumonia with bronchial mucosa involvement is a common finding; however, unlike humans, clinical manifestations from the respiratory tract are rare in dogs with CanL.

## Figures and Tables

**Table 1:** Clinical staging of 31 dogs with leishmaniosis.

Case Number	Leish Stage
1	3 (severe)
5	3 (severe)
11	1 (mild)
12	3 (severe)
15	1 (mild)
19	3 (severe)
21	2 (moderate)
22	3 (severe)
23	3 (severe)
24	4 (very severe)
25	3 (severe)
26	3 (severe)
27	2 (moderate)
30	2 (moderate)
32	1 (mild)
35	3 (severe)
36	3 (severe)
37	2 (moderate)
39	2 (moderate)
40	2 (moderate)
41	2 (moderate)
42	2 (moderate)
43	2 (moderate)
44	2 (moderate)
45	3 (severe)
46	3 (severe)
49	2 (moderate)
51	2 (moderate)
52	3 (severe)
53	2 (moderate)
54	2 (moderate)

**Table 2:** Results of various diagnostic techniques (cytology, immunofluorescence antibody test (IFAT), histopathology, immunohistochemistry, immunocytochemistry) on different samples (lymph nodes, bone marrow, bronchoalveolar lavage fluid (BALF), lung fine-needle aspiration (FNA), serum, bronchial mucosa biopsies) of 31 dogs with leishmaniosis.

Case Number	Lymph Node FNA Cytology	Bone Marrow Cytology	BALF Cytology	Lung FNA Cytology	Serum IFAT Titer	BALF IFAT	Bronchial Mucosa Histopathology	Bronchial Mucosa Immunohistochemistry	BALF Immunocytochemistry	Lung FNA Immunocytochemistry
1	Positive	Positive	Negative	Negative	1/1600	Positive	Negative	Positive	Positive	Positive
5	Positive	Positive	Negative	Negative	1/400	Positive	Negative	Positive	Positive	Positive
11	Positive	Negative	Negative	Negative	1/100	Positive	Negative	Positive	Positive	Positive
12	Positive	Positive	Negative	Negative	1/1600	Positive	Negative	Negative	Positive	Positive
15	Positive	Positive	Negative	Negative	1/3200	Positive	Negative	Positive	Positive	Positive
19	Positive	Positive	Negative	Negative	1/1600	Positive	Negative	Positive	Positive	Positive
21	Positive	Positive	Negative	Negative	1/3200	Positive	Negative	Negative	Positive	Positive
22	Positive	Positive	Negative	Negative	1/1600	Positive	Negative	Positive	Positive	Positive
23	Positive	Positive	Negative	Negative	1/800	Positive	Negative	Positive	Positive	Positive
24	Positive	Positive	Negative	Negative	1/1600	Negative	Negative	Positive	Positive	Positive
25	Positive	Positive	Negative	Negative	1/3200	Positive	Negative	Positive	Positive	Negative
26	Positive	Positive	Negative	Negative	1/3200	Positive	Negative	Positive	Positive	Positive
27	Positive	Positive	Negative	Negative	1/3200	Negative	Negative	Positive	Positive	Negative
30	Positive	Positive	Negative	Negative	1/1600	Negative	Negative	Positive	Positive	Positive
32	Positive	Negative	Negative	Negative	1/1600	Negative	Negative	Positive	Positive	Positive
35	Positive	Positive	Positive	Negative	>1/3200	Positive	Negative	Positive	Positive	Positive
36	Positive	Positive	Negative	Negative	1/3200	Negative	Negative	Negative	Negative	Positive
37	Positive	Positive	Negative	Negative	1/800	Negative	Negative	Positive	Positive	Positive
39	Positive	Positive	Negative	Negative	1/1600	Negative	Negative	Positive	Positive	Positive
40	Positive	Positive	Negative	Negative	1/800	Negative	Negative	Positive	Positive	Positive
41	Positive	Positive	Negative	Negative	>1/3200	Negative	Negative	Negative	Positive	Positive
42	Positive	Positive	Negative	Negative	>1/3200	Positive	Negative	Positive	Positive	Negative
43	Positive	Positive	Negative	Negative	1/3200	Negative	Negative	Positive	Positive	Positive
44	Positive	Positive	Negative	Negative	1/800	Negative	Negative	Positive	Positive	Positive
45	Positive	Positive	Negative	Negative	1/3200	Negative	Negative	Positive	Positive	Positive
46	Positive	Positive	Negative	Negative	1/1600	Negative	Negative	Positive	Positive	Positive
49	Positive	Positive	Negative	Negative	1/3200	Positive	Negative	Positive	Positive	Negative
51	Positive	Positive	Negative	Negative	1/3200	Negative	Negative	Positive	Positive	Positive
52	Positive	Positive	Negative	Negative	1/1600	Positive	Negative	Positive	Positive	Positive
53	Positive	Positive	Negative	Negative	1/800	Negative	Negative	Positive	Positive	Positive
54	Positive	Positive	Negative	Negative	1/200	Negative	Negative	Positive	Positive	Positive

**Table 3:** Bronchoscopic lesions of 31 dogs with leishmaniosis.

Bronchoscopic Lesions	*n* (%)
Edema	15 (48.4)
Hyperemia	7 (22.6)
Mucosal granularity	7 (22.6)
Secretions	2 (6.5)

**Table 4:** Categorization of 19 dogs with leishmaniosis and bronchoscopic lesions.

Groups	*n* (%)
Mild	17 (89.5)
Moderate	2 (10.5)
Severe	0

**Table 5:** Histopathological findings of 31 dogs with leishmaniosis.

Histopathological Findings	*n* (%)
Macrophage infiltration	17 (54.8)
Edema	12 (38.7)
Neutrophilic infiltration	9 (29)
Fibroblasts	7 (22.6)
Lymphocytic infiltration	6 (19.3)
Bronchial gland hyperplasia	6 (19.3)
Thickening of vessel walls	5 (16.1)
Thickening of the bronchial smooth muscles	3 (9.7)

**Table 6:** Categorization of 24 dogs with leishmaniosis and pulmonary histopathological lesions.

Severity of Histopathological Lesions	n (%)
Mild	19 (79.2)
Moderate	5 (20.8)
Severe	0

## Data Availability

Not applicable.
